# Precision and pitfalls: evolving role of ultrasound-guided nerve blocks in Orthopedic perioperative pathway—a perspective

**DOI:** 10.3389/fmed.2026.1806545

**Published:** 2026-04-07

**Authors:** Rui-tao Li, Wei-rong Ren

**Affiliations:** Department of Anesthesiology, The First Hospital of Yulin, Yulin, China

**Keywords:** enhanced recovery after surgery, multimodal analgesia, orthopedic surgery, patient outcome, perioperative care, ultrasound-guided regional anesthesia

## Abstract

Ultrasound-guided regional anesthesia (UGRA) has become an integral part of enhanced recovery protocols in orthopedic surgery, advancing from landmark-based methods to image-guided precision. Yet its broad implementation and comprehensive integration into perioperative care remain challenging. This perspective article argues that while UGRA improves anatomical targeting, intraoperative stability, and postoperative recovery, it also introduces specific risks related to technique, judgment, and complications. A structured, evidence-informed framework is therefore needed to maximize its benefits while proactively mitigating these risks. By scrutinizing current evidence and clinical experience, this article presents a multi-level analysis spanning diverse surgical settings-from trauma to elective procedures-and explores emerging directions in technology, training, and outcomes research. In summary, we propose a “precision with prudence” approach that combines standardized protocols with individualized care. Strengthening multidisciplinary coordination and perioperative continuity can help transform UGRA from a procedural skill into a central element of patient-centered recovery, ultimately improving functional outcomes and long-term quality of life after orthopedic surgery.

## 1 Introduction

### 1.1 Evolution of perioperative management in orthopedic Surgery

Perioperative management in orthopedic surgery has undergone a significant paradigm shift from a “surgery-centric” to a “patient-centered” approach (1). Traditionally, the focus was predominantly on the surgical procedure itself, with postoperative recovery often considered a passive phase. The integration of enhanced recovery after surgery (ERAS) principles has fundamentally redefined this pathway (1). ERAS emphasizes reducing surgical stress and preserving physiological function through evidence-based interventions aimed at shortening recovery time and improving patient outcomes (2). This evolution demands a redefined role for anesthesia: it is no longer confined to providing intraoperative analgesia and unconsciousness but now serves as an active, comprehensive perioperative strategy that facilitates recovery (2). Within this framework, multimodal analgesia has become essential, combining techniques and medications with different mechanisms to enhance pain relief while minimizing side effects (3). Regional anesthesia plays a central role in this strategy due to its effective, targeted analgesia and favorable physiological profile, making it a cornerstone of modern ERAS protocols (3, 4).

### 1.2 Technological revolution of ultrasound-guided regional anesthesia (UGRA)

The practice of regional anesthesia has evolved from reliance on anatomical landmarks and patient feedback to real-time, image-guided precision (5). In traditional practice, peripheral nerve blocks were predominantly performed using landmark-based techniques, in which needle insertion depended on surface anatomical references and clinician experience (6). Although widely adopted, these approaches are inherently constrained by interindividual anatomical variability and the absence of direct visualization, thereby increasing the risk of vascular puncture, nerve injury, and incomplete or failed blockade (7). The subsequent introduction of nerve stimulator-guided techniques represented a partial advancement by enabling indirect localization of target nerves through elicited motor responses (8); however, these methods still lack real-time visualization of surrounding anatomical structures and local anesthetic distribution (5). Collectively, these limitations underscore the need for more precise, reliable, and safety-oriented guidance modalities.

Against this backdrop, the introduction of UGRA represents a transformative advancement. UGRA enables direct visualization of target nerves, adjacent anatomical structures, needle progression, and local anesthetic spread (5). This visual accuracy is supported by robust evidence demonstrating that UGRA increases block success rates, improves precision, and reduces complications such as vascular injury, intraneural injection, and systemic local anesthetic toxicity (8–10). Consequently, orthopedic anesthesia has transitioned from an experience-dependent techniques—primarily referring to landmark-based techniques without direct visualization and, to a lesser degree, nerve stimulator-guided methods that depend on indirect functional responses rather than real-time anatomical imaging—into a reproducible, image-guided discipline. This shift enhances both procedural safety and consistency while minimizing the uncertainties inherent in conventional blind techniques.

### 1.3 Scope and perspective

This study is presented as a perspective article that scrutinizes existing evidence and clinical insights to examine the evolving role, limitations, and future directions of UGRA in orthopedic perioperative care. Rather than providing a systematic review or procedural guideline, the purpose of this perspective article is to offer a conceptual and integrative analysis of how UGRA is reshaping contemporary perioperative strategies.

Central to this perspective is the premise that UGRA offers a superior safety profile compared with conventional techniques, thereby supporting its broader integration into orthopedic ERAS protocols (11). Through improved anatomical precision, reduced complication rates, and enhanced perioperative outcomes, UGRA extends beyond a technical innovation to function as a strategic enabler of patient-centered recovery pathways (8). Its incorporation into ERAS frameworks supports the delivery of high-quality, efficient, and individualized care, thereby reinforcing the overarching goals of modern perioperative medicine.

**Figure 1:**
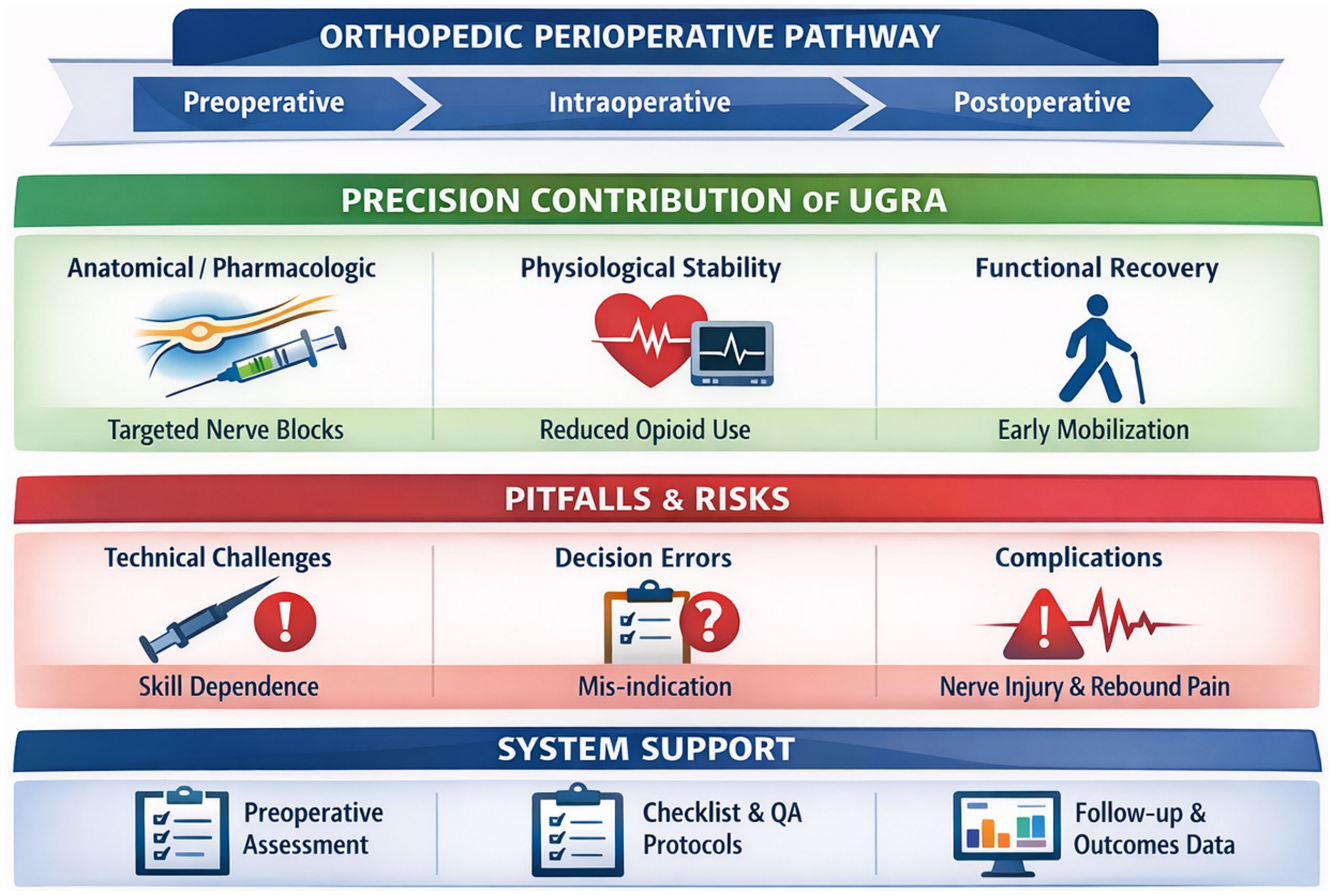
Integrated precision-risk balanced framework of UGRA across the orthopedic perioperative pathway.

Within this framework, UGRA is examined not as an isolated technical intervention, but as an integral component within the continuum of orthopedic perioperative care. It focuses on the systemic role of UGRA in connecting and optimizing preoperative, intraoperative, and postoperative phases. Furthermore, this article adopts a critical perspective by addressing potential limitations, including over-reliance on imaging at the expense of holistic assessment, and the need to align technical performance with meaningful patient-centered outcomes. Finally, we propose an evidence-based, multidisciplinary framework for integrating UGRA into enhanced recovery pathways, with the goal of improving both anesthetic quality and postoperative recovery in orthopedic surgery. The overall conceptual structure of this integration is illustrated in Figure 1.

### 1.4 Literature identification approach

To support the conceptual framework of this perspective article, a targeted literature search was conducted in PubMed and Web of Science. The search strategy employed combinations of key terms such as “ultrasound-guided regional anesthesia,” “peripheral nerve block,” “orthopedic surgery,” “perioperative analgesia,” and “enhanced recovery.” Priority was given to peer-reviewed randomized controlled trials, systematic reviews, consensus guidelines, and large-scale observational studies, thereby strengthening the reliability and clinical relevance of the synthesized insights.

It should be noted that, given the perspective nature of this article, the literature selection process is intentionally presented in a descriptive and narrative manner rather than adhering to a formal PRISMA-based systematic framework. Accordingly, the selected articles were synthesized narratively to highlight major trends, clinical implications, and emerging challenges associated with incorporating ultrasound-guided regional anesthesia into orthopedic perioperative care pathways. To enhance transparency while maintaining methodological appropriateness for a perspective article, Supplementary Figure 1 is presented as a conceptual illustration of the literature identification process rather than a formal PRISMA flow diagram.

## 2 Dimensions of precision: how UGRA reshapes the perioperative pathway in orthopedics

The clinical integration of UGRA represents a transformative shift in orthopedic perioperative care, advancing practice from an experience-based model toward a precision-driven paradigm. By enabling targeted interventions across multiple perioperative stages, UGRA comprehensively optimizes patient pathways to improve clinical outcomes. Its impact can be scrutinized through three interconnected dimensions of precision: anatomic-pharmacologic, intraoperative, and postoperative.

### 2.1 Anatomic and pharmacologic precision

UGRA achieves a fundamental shift from landmark-based approximation to image-guided targeting (12). Direct ultrasound visualization allows clear identification of neural structures and adjacent anatomy while permitting real-time observation of needle placement and local anesthetic dispersion (12). This anatomic accuracy facilitates precise pharmacologic titration. Clinicians can individualize the choice of local anesthetic—selecting specific agents, concentrations, and volumes—based on surgical requirements, anticipated duration, and postoperative analgesic goals (8). For example, a lumbar plexus or femoral nerve block provides targeted analgesia for major lower limb surgery, whereas a focused, low-volume brachial plexus block may suffice for distal upper extremity procedures (13). This tailored approach—spanning fascial plane blocks to selective nerve plexus blocks—ensures optimal alignment with surgical needs, enhancing analgesic efficacy while minimizing excessive motor blockade and related complications (14).

### 2.2 Precision in intraoperative management

UGRA contributes meaningfully to intraoperative physiology and pharmacodynamics. By establishing profound regional analgesia, it substantially reduces the need for systemic opioids and volatile anesthetics (15). This effect results not only from analgesic substitution but also from attenuation of surgical stress, which promotes hemodynamic and metabolic stability (16). In lower limb and truncal surgery, sympathetic blockade induced by UGRA can improve peripheral perfusion and mitigate stress-induced hypertension, supporting intraoperative cardiovascular homeostasis (17). Furthermore, controlled motor blockade improves surgical conditions by enhancing muscle relaxation and patient tolerance to tourniquet ischemia, potentially reducing sedation requirements and facilitating smoother emergence from anesthesia (18).

### 2.3 Precision in postoperative recovery

UGRA plays a pivotal role in modern postoperative recovery protocols. It operationalizes the principle of preemptive analgesia by establishing neural blockade prior to surgical incision, thereby modulating nociceptive signaling and reducing central sensitization (19). This foundational analgesic strategy produces a significant opioid-sparing effect, which in turn lowers the incidence of opioid-related adverse events such as nausea, respiratory depression, ileus, and delirium (20, 21). Most importantly, effective and sustained pain control enables early mobilization—a critical determinant of functional recovery after orthopedic surgery (22). By facilitating timely physical therapy and ambulation, UGRA supports shorter hospital stays, improved joint function, and enhanced long-term patient satisfaction, thereby closing the loop from intraoperative analgesia to functional rehabilitation (20).

## 3 Identifying pitfalls: challenges and limitations in UGRA integration

While the comprehensive integration of UGRA into orthopedic perioperative care offers clear benefits for precision management, it also introduces specific challenges that warrant careful consideration (23). These issues extend beyond technical execution to include clinical judgment, risk assessment, and systemic implementation (12, 24). Recognizing and addressing these limitations methodically is essential for advancing the safe and effective adoption of UGRA in clinical practice.

### 3.1 Technique-related pitfalls

The effectiveness of UGRA relies substantially on operator proficiency, which is associated with a distinct learning curve (25). Mastery requires not only competency in ultrasound imaging but also a thorough understanding of sonoanatomy and hand-eye coordination (25). This dependence on skill contributes to variability in practice standards across different clinical settings (26, 27). Furthermore, ultrasound imaging is subject to interpretive challenges (28). Artifacts—such as acoustic shadowing or reverberation—and anatomical variations can complicate the accurate identification of structures, potentially leading to procedural error. An additional concern is the “visualization paradox,” wherein confidence derived from real-time imaging may inadvertently cause operators to overlook foundational safety measures, including needle aspiration before injection (29).

### 3.2 Clinical decision-making pitfalls

The decision to apply UGRA should be guided by clinical indication rather than technical availability alone (30). A common error is selecting a block that does not align with the surgical site or anticipated postoperative needs, which can lead to inadequate analgesia or unnecessary neurological exposure (31). In trauma patients, UGRA requires heightened caution due to factors such as uncertain anatomy, potential hemodynamic instability, or underlying coagulopathy—each of which can alter the risk–benefit assessment (32, 33). In elective orthopedic settings, there is also a risk of overutilization, where technically feasible blocks are performed without clear evidence of added value over simpler, systemic analgesic options (30).

### 3.3 Complications and risk management

Although UGRA enhances procedural safety, it does not eliminate all risks and may introduce specific complications. Prevention and management of Local Anesthetic Systemic Toxicity remain critical, with current guidelines emphasizing early recognition and prompt administration of lipid emulsion therapy (34). The risk of nerve injury, while reduced under ultrasound visualization, persists through mechanisms such as intraneural injection, drug toxicity, or mechanical compression (35). Additionally, clinicians should remain vigilant for other potentially serious complications, including hematoma formation—particularly in anticoagulated patients—local infection, and complications related to indwelling catheters (36, 37). Structured protocols for prevention, monitoring, and management are necessary to mitigate these risks (34, 36, 37).

## 4 From trauma to elective surgery: differentiated integration strategies in orthopedic contexts

The effective implementation of UGRA in orthopedic perioperative care requires tailored strategies that reflect the distinct clinical demands of different surgical settings (38). The fundamental differences between emergent trauma management and elective surgery—in terms of patient physiology, therapeutic priorities, and time constraints—directly shape the role, execution, and safety considerations of UGRA (39, 40). Therefore, a flexible and context-sensitive framework is essential for its successful integration across varied clinical pathways. Representative context-specific strategies are summarized in Table 1.

**Table 1:** Context-specific strategies for UGRA integration across orthopedic surgical settings

Surgical context	Clinical priority	Preferred UGRA approach	Key benefits	Major concerns
Emergency trauma	Rapid analgesia, stabilization	Single shot/ fascial plane	Fast, opioid sparing	Hemodynamic risk
Elective arthroplasty	ERAS, mobilization	Motor-sparing blocks	Rehab friendly	Over-standardization
Spine/complex	Severe pain, long duration	ESP/continuous catheter	Sustained analgesia	Anticoagulation

UGRA, ultrasound-guided regional anesthesia; ERAS, Enhanced Recovery After Surgery; ESP, erector spinae plane.

### 4.1 UGRA in the emergency trauma pathway

In the trauma setting, UGRA should be applied under significant time pressure, with the primary aim of providing rapid and effective analgesia to facilitate patient evaluation, transport, and subsequent treatment (32, 41). Decision-making should prioritize straightforward, reliable, and rapid-onset techniques, such as single-injection nerve blocks or fascial plane blocks (41, 42). For hemodynamically unstable patients, careful consideration is required to balance the potential vasodilation from sympathetic blockade against the risks of systemic opioid administration (32, 38). In polytrauma cases, a clear hierarchy for block selection is necessary—prioritizing regions that impair respiration or cause the most severe pain. Here, UGRA serves as a synergistic component within a multimodal analgesic plan, reducing the need for deep sedation or high-dose opioids during general anesthesia (32).

### 4.2 UGRA in the elective joint surgery pathway

Within elective joint arthroplasty pathways, particularly in ERAS protocols, UGRA is applied in a more comprehensive and standardized manner (43). Evidence-based block protocols should be established—for example, employing an adductor canal block combined with local infiltration for total knee arthroplasty to provide analgesia while preserving quadriceps function (44). With the growth of outpatient and short-stay joint replacement programs, UGRA techniques should align with accelerated discharge goals (45). This may involve using long-acting single-shot blocks or well-managed continuous peripheral nerve catheters suitable for home use (46). A key consideration in elective settings is the long-term functional outcome: the choice of block should balance optimal pain control against the potential impact on early muscle strength recovery and participation in postoperative rehabilitation (44).

### 4.3 Specialized integration in spine and complex orthopedic surgery

While general anesthesia remains standard for most spine surgeries, UGRA can serve as a valuable adjunct (47). For instance, an ultrasound-guided erector spinae plane block can significantly reduce postoperative incisional pain in posterior spinal fusion procedures. In complex, multilevel, or prolonged surgeries—such as oncologic resections or major limb reconstructions—continuous catheter-based techniques become particularly important and require structured protocols for placement, maintenance, and monitoring (48, 49). Additionally, many orthopedic patients, especially those undergoing major joint or spine procedures, receive perioperative anticoagulation (49). Integrating UGRA in this population demands a careful, individualized risk–benefit assessment, adherence to established anticoagulation guidelines, and often a preference for superficial fascial plane blocks to minimize bleeding risk (36). Coordination between anesthesia and surgical teams is essential to time the block appropriately in relation to anticoagulant dosing.

### 4.4 Representative nerve block techniques in major Orthopedic procedures

Beyond conceptual discussions, several ultrasound-guided nerve block techniques have become widely adopted in contemporary orthopedic practice (Supplementary Table 1). In arthroplasty, the adductor canal block is commonly used for total knee arthroplasty because it provides effective postoperative analgesia while largely preserving quadriceps muscle strength, thereby facilitating early mobilization within ERAS pathways (50). For hip arthroplasty, the fascia iliaca block and the pericapsular nerve group block are increasingly utilized to deliver targeted analgesia to the anterior hip capsule and surrounding structures (51, 52). In spine surgery, the ultrasound-guided erector spinae plane block has emerged as a valuable adjunct for thoracic and lumbar procedures. Evidence suggests that this technique can reduce postoperative opioid consumption and improve early recovery profiles (53). In the context of lower limb trauma, regional nerve blocks are frequently employed to provide rapid and effective analgesia in emergency settings (54). Techniques such as the femoral nerve block, fascia iliaca block, and sciatic nerve block are commonly used for femoral fractures, tibial injuries, and related traumatic conditions, facilitating fracture management, imaging procedures, and early stabilization (55).

The incorporation of these procedure-specific techniques into structured perioperative analgesic protocols may improve the precision of pain management and further strengthen the role of UGRA in orthopedic perioperative care.

## 5 Constructing an optimized perioperative pathway: an integrated framework

A comprehensive and integrated framework is essential to fully realize the benefits of UGRA in orthopedic enhanced recovery while minimizing associated risks. This framework should extend beyond the technical procedure itself to encompass the entire perioperative continuum, integrating UGRA within a patient-centered clinical pathway to optimize safety, effectiveness, and efficiency.

### 5.1 Preoperative assessment and patient stratification

The foundation of an effective pathway is a thorough and individualized preoperative evaluation (56). Clinical decision-making should be supported by evidence-based tools, such as algorithms that consider specific patient factors—including anatomical characteristics, surgical requirements, and comorbidities—to determine the appropriateness, type, and alternatives for UGRA (57). Concurrently, structured patient education is crucial to set realistic expectations, explain the procedure and recovery process, and improve adherence (58). Contraindications should be assessed dynamically up until the time of surgery, with particular attention to changes in anticoagulation status, neurological function, or signs of infection that may influence procedural safety (36, 59).

### 5.2 Intraoperative execution and quality assurance

Intraoperative success relies on standardized protocols to ensure consistency and safety. Detailed checklists should guide critical steps, including equipment setup, aseptic preparation, target identification, and injection technique (60). The use of adjunct technologies can enhance precision and reduce risk: nerve stimulation may confirm needle placement when imaging is ambiguous, injection pressure monitoring can help avoid intraneural administration, and Doppler ultrasound assists in identifying nearby vessels (61–63). Effective multidisciplinary communication is also essential, ensuring that the surgical and anesthesia teams share a common understanding of the anesthetic plan and any necessary procedural adjustments (64).

### 5.3 Postoperative handover and transition management

Seamless postoperative care requires proactive planning for the transition from regional to systemic analgesia. The anticipated offset of the nerve block should inform a stepwise analgesic plan, preventing gaps in pain control as the block recedes (65). For patients undergoing ambulatory or short-stay surgery, discharge criteria should include well-controlled pain, stable motor function, and the absence of complications (66, 67). Appropriate home support—such as clear instructions for catheter management, if used, and access to clinical advice—should be provided (48). Finally, comprehensive follow-up to collect outcomes related to pain, function, satisfaction, and complications is necessary to evaluate and continuously improve the clinical pathway (68).

## 6 Future directions and innovation Frontiers

The integration of UGRA into orthopedic perioperative practice is well-established, yet its full potential will be realized through continued innovation. Future progress will likely focus on enhancing precision, incorporating intelligent technology, and adopting more comprehensive approaches to improve patient outcomes and operational efficiency. Key areas of development include technological integration, pharmacological advances, educational transformation, and new research methodologies.

### 6.1 Technological integration and intelligence

The next generation of UGRA will increasingly merge with digital technologies to improve accuracy and safety. Artificial intelligence may be embedded within ultrasound systems to automate structure recognition, guide needle placement, and alert clinicians to potential risks (69). Augmented and mixed reality platforms could overlay ultrasound images directly onto the patient, offering an immersive, real-time anatomical guide that may reduce procedural complexity and improve accuracy (70). Furthermore, integrated sensor systems or quantitative neuro-monitoring could provide objective data on block onset, spread, and duration, enabling more responsive and individualized analgesic management (71, 72).

### 6.2 Advances in precision pharmacology

Pharmacological research is shifting toward longer-acting, safer, and more selective local anesthetics. Innovations include new drug formulations—such as liposomal or polymer-based carriers—designed to extend analgesic duration and support accelerated discharge pathways (73–75). Concurrently, research into adjuvants is evolving to permit more tailored use, potentially informed by patient-specific factors (76). Equally important is the ongoing development of local anesthetics with improved cardiac and neurological safety profiles, which would broaden the applicability of UGRA in higher-risk patients (77).

### 6.3 Transformation of educational systems

As UGRA becomes more technologically advanced, education and training should adapt accordingly. Simulation-based training, coupled with structured competency assessments, can standardize skill acquisition and certification (23). Educational programs should also emphasize multidisciplinary competencies—including ultrasound interpretation, complication management, and perioperative coordination—to enhance team-based care (78, 79). To ensure sustained proficiency, systems for continuous skill maintenance, such as periodic review and digital case-based learning, will be essential (80).

### 6.4 Shifts in research paradigms

Future research should transition from traditional procedural metrics—such as block success—toward comprehensive, patient-centered outcomes. Relevant endpoints include functional recovery, quality of life, incidence of chronic pain, and patient-reported experiences (20, 81). Real-world evidence from large registries and pragmatic studies will be vital to understand long-term safety and effectiveness across diverse clinical settings (82). Additionally, rigorous health economic analyses are needed to quantify the value of UGRA in reducing opioid use, shortening hospital stays, and improving recovery, thereby supporting evidence-based clinical and policy decisions (83).

## 7 Summary

UGRA is a critical enabling technology for advancing orthopedic perioperative care toward greater precision and comprehensive management. Beyond providing effective analgesia, it supports the integration of preoperative, intraoperative, and postoperative phases into a coordinated, patient-centered pathway. Realizing its full potential, however, requires careful attention to its technical, cognitive, and procedural limitations. Successful implementation thus depends on the synergy of three elements: skilled technical execution, evidence-based clinical decision-making, and robust institutional support.

These insights advocate for a “precision with prudence” approach to UGRA. Clinically, this entails combining standardized, evidence-based protocols with individualized patient assessment and planning. Strengthening interdisciplinary communication and continuity of care throughout the perioperative journey is essential to translate technical success into meaningful patient outcomes.

Looking forward, UGRA is poised to evolve from a standalone procedural skill into a core component of integrated perioperative medicine. Through sustained innovation in technology, pharmacology, education, and outcomes-based research, its application can be further refined. The overarching aim remains to improve not only perioperative analgesia but also functional recovery and long-term quality of life, thereby elevating the standard of rehabilitation following orthopedic surgery.

## Data Availability

The original contributions presented in the study are included in the article/Supplementary material, further inquiries can be directed to the corresponding authors.
