# Supplementary figures and images for: Precision and pitfalls: evolving role of ultrasound-guided nerve blocks in Orthopedic perioperative pathway—a perspective
Source: Front Med (Lausanne). 2026 Apr 7;13:1806545. doi: 10.3389/fmed.2026.1806545 (PMC13095673; doi:10.3389/fmed.2026.1806545)

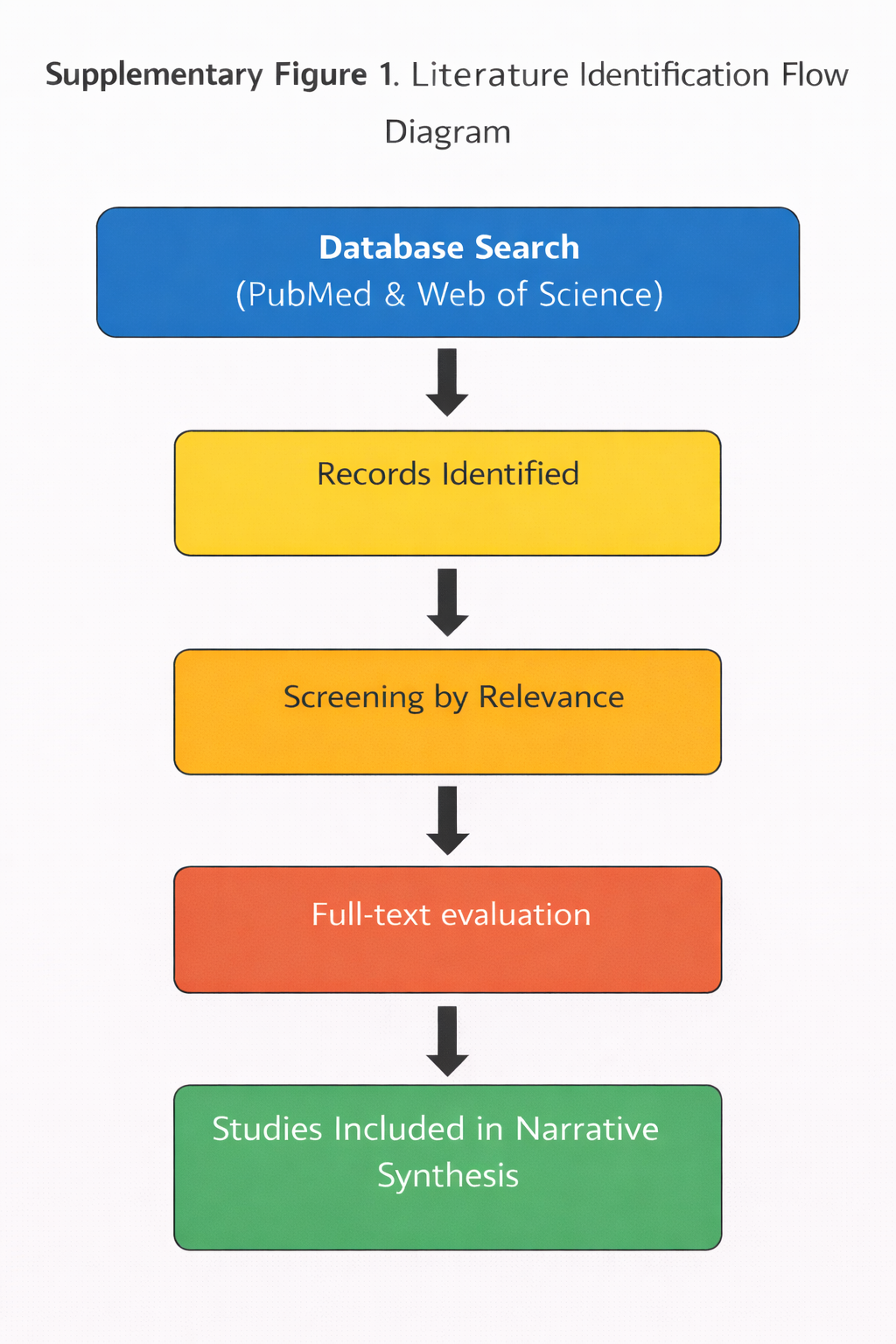

Supplement: Supplementary file 1 [file Image_1.TIFF]
