# Supplementary material for: Precision and pitfalls: evolving role of ultrasound-guided nerve blocks in Orthopedic perioperative pathway—a perspective
Source: Front Med (Lausanne). 2026 Apr 7;13:1806545. doi: 10.3389/fmed.2026.1806545 (PMC13095673; doi:10.3389/fmed.2026.1806545)
Supplement: Supplementary file 2 [file Table_1.docx]

**Supplementary Table 1. Common Ultrasound-Guided Regional Anesthesia (UGRA) Techniques in Orthopedic Surgery**

| **Surgical Context** | **UGRA Technique** | **Target Nerve/**  **Anatomical Region** | **Primary Clinical Purpose** | **Key Clinical Advantage** |
| --- | --- | --- | --- | --- |
| Total Knee  Arthroplasty | Adductor Canal Block | Saphenous nerve within the  adductor canal | Postoperative analgesia | Preserves quadriceps strength, enabling early mobilization within ERAS pathways |
| Total Hip  Arthroplasty | Fascia Iliaca Block | Femoral nerve and lateral  femoral cutaneous nerve | Analgesia for anterior hip and  thigh | Simple technique with broad analgesic coverage |
|  | Pericapsular Nerve  Group Block | Articular branches to the  anterior hip capsule | Targeted hip analgesia | Provides selective analgesia with minimal motor impairment |
| Hip Fracture/  Emergency Trauma | Fascia Iliaca Block | Femoral nerve and lumbar  plexus branches | Rapid analgesia in emergency  settings | Facilitates patient transport and imaging procedures |
|  | Femoral Nerve Block | Femoral nerve | Pain control for femoral shaft  or neck fractures | Effective analgesia with rapid onset |
| Lower Limb  Trauma Surgery | Sciatic Nerve Block | Sciatic nerve (popliteal or  subgluteal region) | Analgesia for tibial or foot  injuries | Provides extensive distal lower limb analgesia |
| Spine Surgery | Erector Spinae  Plane Block | Dorsal rami of spinal nerves | Postoperative analgesia  after spinal surgery | Reduces opioid consumption and improves recovery profiles |
| Major Orthopedic  Reconstruction | Continuous Peripheral  Nerve Block | Target nerve depending  on surgical site | Prolonged postoperative  analgesia | Allows sustained analgesia and supports early rehabilitation |

**Abbreviations**: UGRA, Ultrasound-Guided Regional Anesthesia; ERAS, enhanced recovery after surgery.
